# Galaxy and Apollo as a biologist-friendly interface for high-quality cooperative phage genome annotation

**DOI:** 10.1371/journal.pcbi.1008214

**Published:** 2020-11-02

**Authors:** Jolene Ramsey, Helena Rasche, Cory Maughmer, Anthony Criscione, Eleni Mijalis, Mei Liu, James C. Hu, Ry Young, Jason J. Gill

**Affiliations:** 1 Center for Phage Technology, Texas A&M University, College Station, Texas, United States of America; 2 Department of Biochemistry and Biophysics, Texas A&M University, College Station, Texas, United States of America; 3 Department of Animal Science, Texas A&M University, College Station, Texas, United States of America; Johns Hopkins University, UNITED STATES

## Abstract

In the modern genomic era, scientists without extensive bioinformatic training need to apply high-power computational analyses to critical tasks like phage genome annotation. At the Center for Phage Technology (CPT), we developed a suite of phage-oriented tools housed in open, user-friendly web-based interfaces. A Galaxy platform conducts computationally intensive analyses and Apollo, a collaborative genome annotation editor, visualizes the results of these analyses. The collection includes open source applications such as the BLAST+ suite, InterProScan, and several gene callers, as well as unique tools developed at the CPT that allow maximum user flexibility. We describe in detail programs for finding Shine-Dalgarno sequences, resources used for confident identification of lysis genes such as spanins, and methods used for identifying interrupted genes that contain frameshifts or introns. At the CPT, genome annotation is separated into two robust segments that are facilitated through the automated execution of many tools chained together in an operation called a workflow. First, the structural annotation workflow results in gene and other feature calls. This is followed by a functional annotation workflow that combines sequence comparisons and conserved domain searching, which is contextualized to allow integrated evidence assessment in functional prediction. Finally, we describe a workflow used for comparative genomics. Using this multi-purpose platform enables researchers to easily and accurately annotate an entire phage genome. The portal can be accessed at https://cpt.tamu.edu/galaxy-pub with accompanying user training material.

## Author summary

In the modern genomic era, scientists without extensive bioinformatic training need to apply advanced computational analyses to genome annotation. At the Center for Phage Technology, we use two open source, web-based platforms: Galaxy, for reproducible computational analyses, and Apollo, a collaborative genome annotation editor, to facilitate annotation of phage genomes. Unlike the genomes of most cellular life forms, phage genomes are usually a single contiguous molecule <200,000 bases in length. Their size allows high standards for complete, evidence-based annotations, and is amenable to genomics education settings. The Galaxy and Apollo system described here is used for original biological research and development of new bioinformatic tools to analyze many individual phage genomes, as well as clusters of related phages. Our robust suite of phage-oriented tools moves all aspects of control and choice into the user’s court. In comparison to widely-used automated and fast command-line annotation methods, our integrated and flexible approach benefits from trained human intervention to result in high-quality final annotations. We have educated a steady stream of scientists, including both undergraduate and graduate students, informally and through formal university course offerings using Galaxy-Apollo to annotate phage genomes, deposit the data in public sequence repositories, and publish the results.

This is a *PLOS Computational Biology* Software paper.

## Introduction

Bacteriophage, or phage, are the viruses of bacteria. Their study cracked open critical concepts in genetics, and allowed detailed gene mapping before genome maps could be generated with ease [1,2]. While phage genomes were the first to be sequenced in their entirety, phage research declined considerably before sequencing technologies took off. Researchers from disparate fields in the modern age have come to a new appreciation for the potential that phage have to help solve current problems, as well as the commensurate challenges facing their application [3]. Scientists around the world are collecting phages for a diverse panel of bacterial hosts that are relevant for the clinic, in industry, and as model organisms. One stated intent is to establish organized repositories, or phage banks, as a community resource for their distribution [4,5]. Coupling this with a surge in use of phage for education and research training, an incredible boom of phage sequencing has also emerged, with great promise for extending our understanding of fundamental phage biology [6].

The great sequencing explosion has resulted in many new viral [7] and phage genomes being deposited into online databases. Because these genomes are small, we can have high standards for complete, confirmed contigs, and high-quality structural and functional annotation. Unfortunately, many of the tools available to accomplish this task are command-line based. When tools available for annotation run on the command-line, they are not accessible to many biologists. Even if they are, the output is not visual, integrated, or contextualized, and requires much outside analysis. NCBI has released a suite of command-line interface tools for (eukaryotic) virus annotation recently [8], and new phage-specific annotation pipelines are command-line based making teamwork less fluid [9]. While the capability to do the needed analyses is there, it is still out of reach for many teams. A great need in the field is therefore to have easy-to-use, web-based tools with a graphical user interface for annotation and community annotation platforms to improve quality and promote shared input on phage assessment for use in any given application. Because phages are being rushed into application in humans for phage therapy without measured and thorough safety checks put in place, many others agree that manual inspection for high-quality annotation is needed [10]. The Center for Phage Technology (CPT) at Texas A&M University has harnessed two powerful online platforms to solve this problem.

## Methods

### Galaxy & Apollo at the CPT

**Fig 1:**
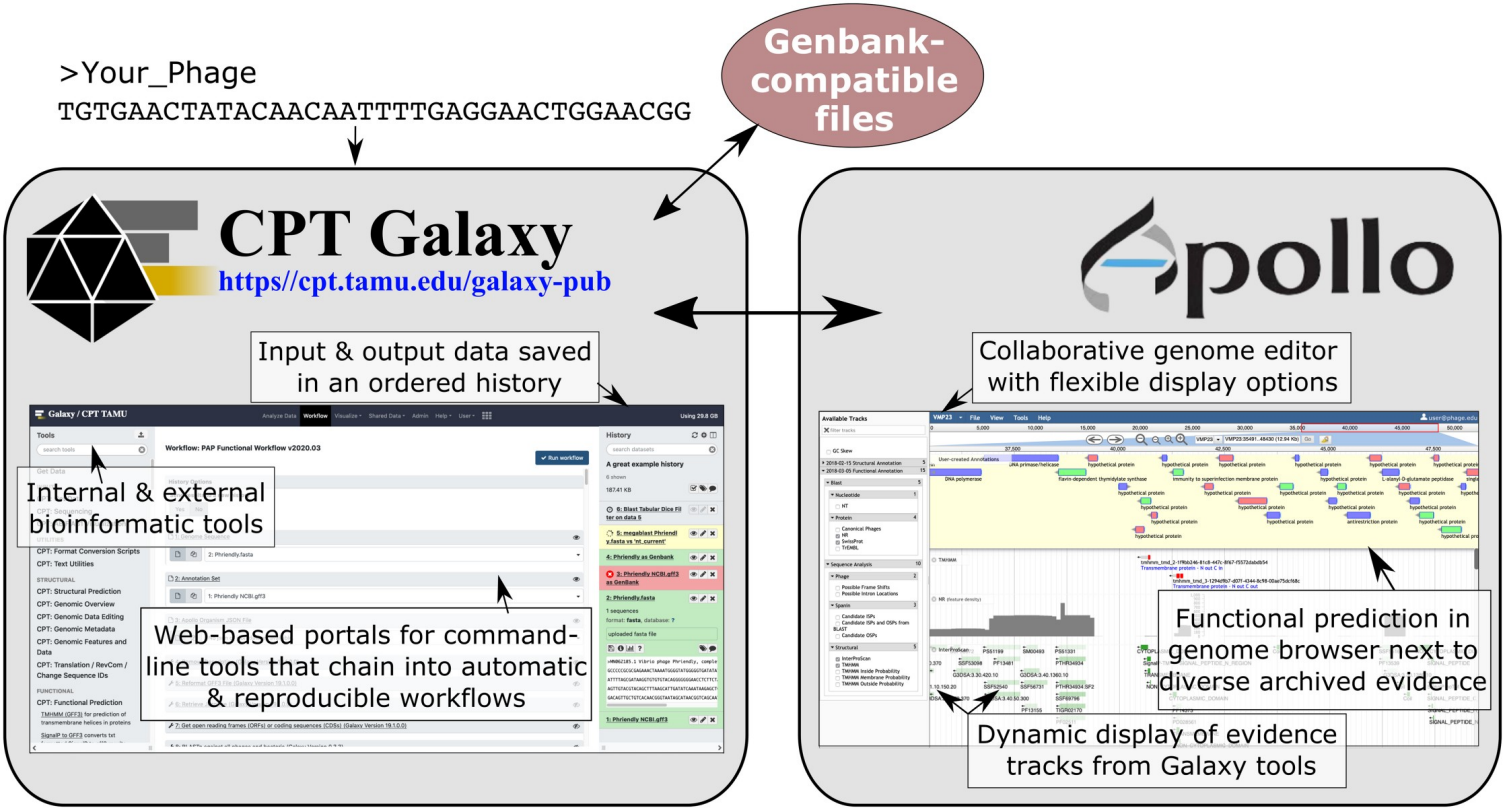
The Galaxy and Web Apollo interface used for analyses and annotation. By coupling the Galaxy platform for analyses with the editing capabilities in Apollo, contextualized evidence can be used to iteratively annotate genomes as a team effort.

The Galaxy Project is a web platform suitable for beginner and advanced biologists to perform analyses on biological sequence data [11]. Apollo is a browser-based, evidence-driven, and community-focused genome annotation editor based on the popular JBrowse genome viewer [12,13]. The CPT created a Galaxy-Apollo bridge to link the powerful sequence analysis capabilities of Galaxy with the evidence-based community annotation platform of Apollo to provide a complete environment for the analysis and annotation of phage genomes (Fig 1) [14]. To date, more than 120 phage genomes have been annotated using this platform and publicly deposited (see NCBI BioProject PRJNA222858). Documentation on the CPT’s Galaxy-Apollo phage annotation pipeline, written at a level suitable for teaching undergraduates, accompanies the CPT resources at https://cpt.tamu.edu/training-material/, with new tutorials added regularly.

### CPT Galaxy, a flexible web-based platform for computational analyses

The major benefit of Galaxy as a bioinformatics platform is that it allows traditionally-trained biologists to perform, record, and visualize complex analyses on their own data within a browser-based graphical user interface, rather than at the command-line. Researchers spend their time analyzing the biological implications of results, rather than troubleshooting the execution of scripts or managing files at the command-line. Crucially, the browser-based interface makes the system accessible to the common scientist. A second powerful feature of Galaxy is the ability to chain tools together in workflows that run jobs (the equivalent of a computational experiment) automatically, which makes the system convenient for programmers and bench scientists alike. Local or worldwide project administrators maintain the system operation, update tools, and provide user support.

Detailed descriptions of Galaxy functionality are provided by the developers, and here we give only a few highlights pertinent to our discussion on its use for phage annotation [11]. The user interface in Galaxy revolves around three main panels where the work is ordered (Fig 1). Tools, or bioinformatic scripts, for data analysis are cataloged and searchable in the left panel. The center Data Analysis panel provides a point-and-click atmosphere for setting job parameters and viewing data. The right-hand panel visualizes the stored history of each user analysis, which enables good documentation and promotes reproducibility. Galaxy histories, akin to a digital notebook, can be shared among users.

The standard Galaxy installation comes with tool sets contributed by the scientific community, and can be expanded through tools available in the ToolShed, by adapting open-source tools for the Galaxy interface, or by writing new scripts to suit specialized analysis needs [15–17]. The CPT Galaxy instance offers various phage-specific tools described below and a comprehensive selection of curated BLAST databases, including SwissProt, NCBI nt and nr, as well as certain custom databases [18]. A common challenge in using a large collection of tools from different sources is the interoperability of the outputs. To facilitate moving data output from one tool into the next, we have also gathered, adapted or produced a comprehensive set of scripts that convert or parse various tool outputs into standardized formats, including conversions between tab-separated value (tsv), XML, Genbank, and GFF3 formats (see S1 Table for partial list, search at https://cpt.tamu.edu/galaxy-pub for full list).

### Apollo at the CPT for teaching and crowd-sourcing advanced phage genome annotation

JBrowse is a widely-used, embeddable genome browser, capable of displaying genome annotations and features from the level of the entire chromosome down to the DNA sequence [19]. Apollo is a web-based genome editor that builds upon JBrowse to provide the ability to do persistent, manual genome annotation and curation inside an internet browser window. Multiple users can view and/or edit the same organism, resulting in Apollo being colloquially referred to as the ‘Google Docs’ for genome annotation. Whole genome and feature analysis results conducted in Galaxy are displayed in tracks below their corresponding locations in the genome, allowing the user to integrate multiple types of evidence within genomic context when making annotations. The centralization of evidence, especially within genomic context, makes the view comprehensive and spares users from mentally integrating evidence in disparate formats from multiple sources, a key achievement of the system. A virtually unlimited number of evidence tracks can be added, dictated primarily by the user’s need for, and access to, additional analysis types. Various forms of metadata can be added in both standard and free-form entry methods, allowing rich annotation of each feature within a genome. As needed, additional evidence from up-to-date analyses in Galaxy can be added to the Apollo organism evidence tracks through the Galaxy-Apollo bridge.

### The Galaxy-Apollo bridge

To meet the needs of reproducible phage annotation and scaling these processes for the influx of new phage genomes, the CPT developed a Galaxy-Apollo bridge. The bridge consists of a number of Galaxy tools which interface directly with Apollo, enabling Galaxy users to move data into Apollo, automate certain tasks within Apollo, and finally to extract user-curated annotations back into Galaxy for further downstream analysis.

During the annotation process, a user will create a JBrowse instance within Galaxy, combining the genome and analysis results for visualization. The **Create or Update Organism** tool sends this data to Apollo either as a newly created organism in the Apollo system, or as an update to an existing one. The **Annotate** tool uses the output of the Create or Update step to provide an iframe window into Apollo, permitting users to retain the context of their Galaxy analysis history and tools, while allowing users to easily switch back to interactive analysis. When manual curation is complete, users can run **Retrieve Data**, to fetch the data from Apollo’s ‘User-created annotations’ track into Galaxy. Tools including **GFF3 to Apollo Annotations** and **Delete all annotations from an Apollo record** enable significantly more automation between Galaxy and Apollo, and future updates are working toward entirely automated annotation pipelines. This set of tools bridges the two different worlds of Galaxy and Apollo and tells one cohesive story: from DNA, to an annotated genome, with the reproducibility of Galaxy and the freedom and real-time collaboration of Apollo.

These tools inspired the Galaxy community to start the Galaxy Genome Annotation project (GGA; https://github.com/galaxy-genome-annotation/), resulting in the development of a new Python library for interacting with Apollo, a command-line suite for Apollo, and a new set of Galaxy tools generated from the Python library.

### Documentation available for new users

To promote widespread adoption by biologists, step-by-step tutorials with background for using Galaxy tools and the Apollo platform are freely available at the CPT website: https://cpt.tamu.edu/training-material/. Their format is based on resources provided by the Galaxy Training Network [20]. Tutorials are written at levels suitable for use by undergraduates learning how to annotate phage genomes. The topics covered make them useful for the scientist as well, including tutorials on phage genome assembly from Illumina reads and instructions for polishing and depositing genomes in the NCBI Genbank database.

### Galaxy tools useful for phage annotation

The customization of the CPT Galaxy instance is best understood as a unified collection of tools that address the specific needs for phage genome annotation; these tools are available for ad hoc usage or as part of a workflow within Galaxy. The challenges associated with phage genome annotation are inextricably linked with general prokaryotic annotation, and intrinsically different from eukaryotic systems. Since intron splicing is rare in prokaryotes and phages, and thus is not assumed *a priori*, tools in the CPT Galaxy are tailored to take advantage of the fact that genes and protein-coding sequences can be called directly from the DNA sequence. Furthermore, many features of the pipeline described below could also be useful for annotating bacterial genomes, although the larger size of bacterial genomes would benefit from increased automation in certain procedures. Below, we describe the development of resources motivated by gaps we experienced within the available annotation tools and platforms.

### Quality control of gene calling using ShineFind

All phages rely on host machinery for translation, and as such their genes typically have recognizable prokaryotic signals. The Shine-Dalgarno (S-D) sequence is a component of the ribosome binding site (RBS) situated upstream of the start codon in prokaryotic genes, and its presence can serve as a strong indicator of gene starts. However, gene callers with high-quality algorithms do not universally include the ribosomal binding site when predicting the locations of protein-coding genes. We wrote a stand-alone tool called **ShineFind** to annotate S-D sequences as part of the standard gene model in Apollo. This Python script accepts GFF3-formatted gene calls and its associated FASTA DNA sequence file as inputs. ShineFind then extracts the upstream sequence for all CDS features that do not already have an RBS in their gene model; this defaults to extraction of upstream nucleotides 3–24, but is editable by the user at runtime. Those upstream sequences are then searched for matches to the *E*. *coli* consensus S-D sequence AGGAGGT, or smaller subsets of this sequence (see full list in S2 Table). By default, the longest match to the consensus is returned, but all the smaller matches can also be output. The returned S-D match is added to the GFF3 gene model as a child of the gene feature and named “Shine_Dalgarno_sequence” (a sequence ontology term [21,22]), and can then be displayed within Apollo. The consensus S-D sequence and recognized subset sequences can be swapped for an alternate sequence set by editing the Python script. GFF3 annotations that already contain S-D annotations can be stripped of these features using the tool **GFF3 Feature Type Filter**, which is also part of the Structural Workflow described below.

### Finding candidate spanin genes

There are special cases among phage genes, such as the lysis genes, which are difficult to detect by standard methods relying on simple sequence similarity. The spanin genes needed for outer membrane disruption during phage lysis of Gram-negative bacterial hosts are often missed or mis-annotated, largely due to their unique genetic architecture in which one gene is often embedded in an alternate reading frame within the sequence of another [23]. Spanins can function as a single or two-protein system, but the proteins share the same characteristics. The inner membrane spanin (i-spanin) contains a transmembrane domain, whereas the outer membrane spanin (o-spanin) will be cleaved and lipoylated by the bacterial lipoprotein processing system at a Cys residue at the end of a lipobox motif [24,25]. Their varied genetic architectures, particularly when the o-spanin gene is fully embedded within the i-spanin gene, and clear domain signatures are keys to the suite of tools that we have developed to find them [26].

To account for the fact that spanin genes are highly prone to be uncalled or called with incorrect start sites, spanin-finding begins with naïve ORF calling by the tool **Get open reading frames (ORFs) or coding sequences (CDSs)** [16,17] finding all possible ORFs (using the NCBI translation table 11 for Bacteria and a 30 aa minimum length cutoff), and generating an output in GFF3 format. The gene model within the GFF3 file is corrected by the addition of a gene parent feature to every CDS. The ORFs are then filtered to include only those with a common phage start codon (ATG, GTG or TTG) and ShineFind is used to add potential Shine-Dalgarno sites where they exist.

From there, the naïve gene sequences are translated and analyzed in three ways. 1) Protein similarity to spanins in a curated database [23] is determined by BLASTp with a 0.001 expectation value cutoff. 2) Potential lipobox motifs expected for outer membrane spanins are identified using the tools **LipoP** [27] and **Identify Lipoboxes**, a less stringent regex amino acid motif search for the four-residue motif [ILMFTV]-[any residue except REKD]-[GAS]-[C], or [A]-[W]-[AGS]-[C]. 3) Inner-membrane spanin candidates are identified through **TMHMM**, which predicts transmembrane regions [28]. Finally, to reduce noise, the results from Lipobox/LipoP and TMHMM predictions are cross filtered to require putative inner- and outer-membrane spanin gene pairs occur within 50 nucleotides of each other and be located on the same strand, consistent with the architecture of known spanin genes [23]. Analysis using both explicit protein similarity by BLASTp and searching for more general spanin signatures allows for the identification of all spanin types and high-quality selection of likely start sites. This approach was able to identify a test set of experimentally verified spanins. However, the output needs to be critically assessed before genes are confidently assigned spanin functional predictions; the signature-based approach of identifying genes that pair with nearby genes containing lipoprotein or TMD signals is prone to producing multiple false-positive results in the average phage genome, as it ignores genomic context and the presence of other genes. These false-positive results can typically be excluded upon cursory examination of the results due to their presence embedded within other, obviously non-spanin genes. Additionally, drawing connections within novel phages has allowed the computational identification of novel spanin genes [23].

### Identifying interrupted genes

Several types of interrupted genes are present in phages, including programmed translational frameshifts, introns or inteins. Researchers typically use an integrated approach to detect the presence and boundaries of expected interrupted genes. Because the functional analysis performs sequence similarity searches, we can take advantage of those results to identify interrupted proteins compared to their uninterrupted counterparts present in the database. Due to biological variety in this area and chain annotation that tends to propagate common annotations (over quality annotations), much work is needed here to populate the databases with accurate annotations. The **Intron Detection** tool can detect legitimate introns (often in essential genes), frameshifts or premature stops produced by sequencing errors (such as those introduced by long-read sequencers), programmed translational frameshifts (frequently found in capsid proteins or tape measure chaperones), and other split or duplicate gene phenomena.

In the CPT Galaxy pipeline, the interrupted genes detection tool parses XML-formatted BLASTp results to determine whether multiple query proteins from the phage genome have amino acid identity to a single target protein in the database. The presence of a single database protein as multiple discrete segments in the query genome is the signature of a gene disrupted by an intron, programmed translational frameshift, or sequencing artifact. The tool requires that the separated gene fragments in the query genome exist within a user-defined number of bases, with the default set at 10,000. Separation minimum evaluates overlap of queries on the subject sequence (in case gene calls with incorrect starts were input); negative values allow overlap and positive/zero values allow separation. Logical checks in the script include removing alignments with high identity over a short part of a longer segment (identities/hsp length<0.3 are discarded), requiring at least two unique high scoring pairs for any given target, and ensuring that the common hits are encoded on the same strand. This tool can also detect separated genes that span the genome ends, for cases where a genome may circularize or if it is opened in the middle of a gene.

In the case of introns, which are self-splicing RNAs usually found in essential genes, they share common secondary RNA structure but are not commonly predicted as part of annotation pipelines unless they contain a well-known intron-encoded protein. Studies have shown that ~25% of bacteria contain genes with group II introns, while phage regularly contain group I introns [29–31]. In the case of T4 and T4-like phages, these mobile elements are seen to vary substantially [32–34]. The most reliable way to find the true boundaries is by manual inspection of high-quality alignments of the intron-disrupted phage protein segments against a non-interrupted homolog from the database, which is identified in the tool output in Apollo. When uninterrupted analogs do not exist in the database, or the nearest uninterrupted analog has weak sequence identity, boundaries cannot be accurately determined bioinformatically.

There are several kinds of important proteins in phages that are produced via programmed translational frameshifting mechanisms, such as the capsid or tail proteins and the tail tape measure chaperones, of which λ is the best characterized example [35–40]. When the frameshifted downstream open reading frame of a protein has been called as a separate protein-coding gene (often without a strong SD), it can be detected by the interrupted genes tool. However, in cases where no experimental data exists for the matched protein in the database, the potential for an interrupted gene should be interpreted with caution.

Finally, the interrupted genes tool can be very helpful for interpreting long reads with many errors, such as those that result from nanopore sequencing. Taking into consideration the noisy output of gene calling (reducing stringency for ORF length and starts), this will be most successful where the genome has high similarity to previously deposited genomes.

### Custom BLAST databases and BLAST restriction by TaxID

The mainstay method for sequence comparison in genome annotation is BLAST [41]. While the number of sequenced phage genomes present in the NCBI database has increased rapidly since next-generation sequencing became widespread [42,43], so have sequences of cellular organisms and eukaryotic viruses [44]. Sequences outside bacteria and phages are usually not relevant to our analyses, drown out low similarity hits of interest, and significantly increase required processing time. The CPT Galaxy instance, with locally installed database copies updated about three times per year from their respective home sites, offers at least two ways to mitigate those weaknesses: custom databases and restriction by TaxID.

*Custom databases*. Users can generate their own local databases in Galaxy (via the **NCBI BLAST+ makeblastdb** tool), or use public databases pre-compiled by the CPT. Significantly, we offer a ‘Canonical Phages Database’, representing the proteomes of phages with significant publication history or of historical importance. This list includes paradigm phages such as Lambda, T4, and T7, as well as P22, P2, and phi29 (see S3 Table for full list). The reason for generating this custom database is to ensure that those phage proteins annotated based on experimental evidence are detectable as hits against novel phage proteins, rather than them being lost amid a slew of less relevant results. This ensures that when there is a single degree of separation between the predicted protein of a novel phage and a studied protein in a canonical phage, that prediction can have higher confidence.

When analyzing phage genomes for therapy applications, toxin/anti-toxin systems, restriction-modification systems, CRISPR-related proteins, and anti-CRISPR proteins are of particular interest to detect. Despite wet bench experiments being performed on many of these proteins, they are poorly annotated in most databases. Custom databases from users or the community can be added to Galaxy for specialized analyses.

*Restriction by TaxID*. In the **BLAST+ 2.9.0** suite using version 5 databases, standalone BLAST jobs can now restrict searches by TaxID, as has long been available on the NCBI BLAST webserver. On the CPT Galaxy instance, we modified the BLAST wrapper to make this functionality available, with searching using non-species-level IDs available with the TaxonKit tool to retrieve all species-level TaxIDs that are required by BLAST 2.9 [45]. Using curated lists of TaxIDs that include all phages, bacteria, and/or canonical phages (S3 Table), we are able to drastically reduce the processing time while targeting searches more effectively.

### Additional tools

In CPT Galaxy, there are also assembly tools, including **SPAdes** and **FastQC**. Since phage genomes are relatively small, typically <200 kb [46], they usually assemble completely in a single contig that is opened randomly by the assembler. Biologically, phage genomes can have physical ends with or without terminal repeats, or be circular [43]. As with other genome browsers, wraparounds are not supported at this time in Apollo. Therefore, the genome is displayed linearly and users must exercise careful judgement on interpretation of all features that border or span the boundary of unannotated genomes. Relatively few phages have had the state of their physical genomic termini probed. Therefore, we rely on comparisons to phages with empirically verified ends and computational predictions, whose accessibility is limited by the specific sequencing technology used. **PhageTerm** is an end prediction program installed in CPT Galaxy [47]. These software assessments can be useful in determining the most logical place to re-open a genome, usually decided by convention in the field. **Genome Editor** is a tool that allows rearrangement of the genome, with or without associated annotations. There are also tools that extract subsets of data from various genome record file types, validators to check their format, and a **PhageQC** report generator, useful for reviewing the quality of external genome annotations. For those accustomed to or desiring an external tabular file that collates their annotation data, the **Annotation Table** tool generates a downloadable Excel-compatible table. These, and a whole suite of plain text, table, and protein/nucleic acid manipulation tools are searchable at https://cpt.tamu.edu/galaxy-pub/.

After all desired analyses are completed, the logical next step is to deposit the sequence with its annotations into one of the public sequence repositories. The tools to retrieve the data from Apollo into Galaxy, as well as interconvert between file types, aid in this essential step. The **Retrieve Data** tool yields a DNA FASTA file and GFF3 file. These can be converted into a Genbank file with the bacterial (default) translation table qualifier added using the **GFF3 to Genbank** tool, then into the 5-column table used with NCBI’s BankIt service using the **Genbank to Five Column Format** tool. Updates and new tools are regularly added to the CPT Galaxy platform. The most current development version is available through our public Github at (https://github.com/TAMU-CPT/galaxy-tools).

### Workflows in Galaxy facilitate efficient multi-step analyses required for phage genome annotation

While having all these tools in one place is useful, the human hours required to sit at the computer and manually move the output from one tool into the next, and converting those files into compatible formats along the way, is prohibitive. We prefer to spend our time on the things that computers cannot do as well: making judgment calls on the annotations. Galaxy workflows offer the ability to chain many tools together automatically, such that a long and tedious analysis can be reproducibly applied to multiple datasets with a few clicks. Three main workflows are the mainstay of our phage annotation pipeline: the Structural Annotation workflow, the Functional Annotation workflow, and the Comparative Genomics workflow. Using the tools described above and in S1 Table, analysis is conducted in Galaxy and then transferred to Apollo via the Galaxy-Apollo bridge, where evidence is displayed for interrogation by the user, who has ultimate control over the annotation process.

### Structural annotation workflow

**Fig 2:**
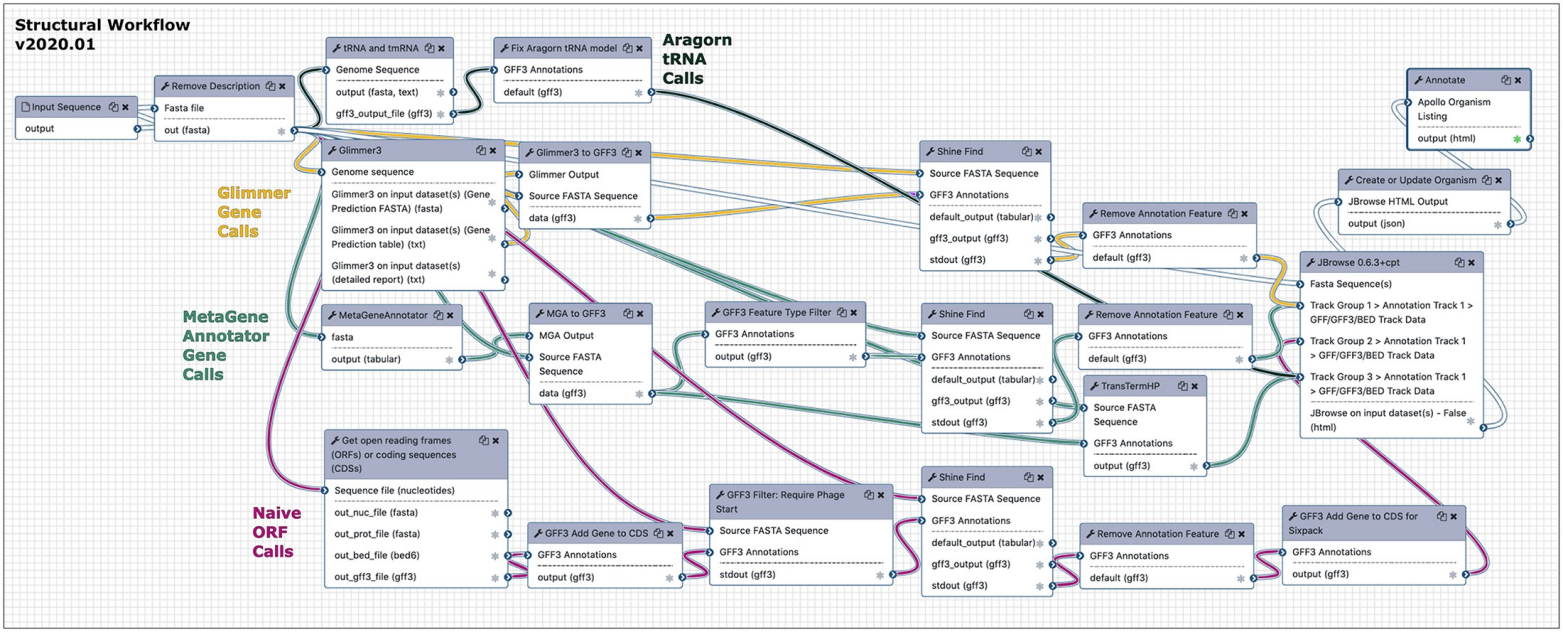
The structural workflow chains together tools in Galaxy for gene calling. The structural workflow accepts as input a nucleotide FASTA genome sequence, and processes it through ARAGORN for tRNAs (dark noodles), Glimmer and MetaGeneAnnotator for high-confidence gene predictions (golden and teal, respectively), and Get ORFs as a naïve ORF/CDS caller (magenta). Potential protein-coding genes are filtered to ensure the presence of a phage (ATG/GTG or TTG) start codon and a Shine-Dalgarno feature is added to all features that have a detectable match. These are interconverted between formats and the gene models are corrected for display in Apollo.

The purpose of the structural workflow is to provide the researcher with the evidence needed to make high-quality, confident gene calls with accurate prediction of the gene start. This is accomplished in 23 total steps from input genome FASTA file to the updated Apollo organism with evidence tracks (Fig 2). The structural workflow is relatively quick for phage genomes, usually taking <20 minutes to complete.

From the naked input DNA sequence, five separate gene analysis tracks are initiated. Two gene callers, **GLIMMER** 3.0 and **MetaGeneAnnotator** v1.0 (MGA), both popular prokaryotic prediction programs, are used to predict the locations of protein-coding genes [48,49]. Their outputs are converted into GFF3 format and processed by ShineFind; RBS sequences assigned by MGA are discarded (feature filter step) in favor of the ShineFind algorithm. The outputs are then properly formatted with a gene model structure that follows gene-CDS and gene-Shine_Dalgarno parent-child relationships for display as a feature track for JBrowse. The MGA gene calls are used to predict rho-independent transcriptional terminators using **TransTermHP** [50]. A third gene caller, **Get open reading frames (ORFs) or coding sequences (CDSs)**, uses the Sixpack framework from EMBL, preset to use the NCBI translation table 11, for minimum 30 aa long ORFs with both a start and stop codon [16,17]. The GetORFs output is further filtered to only genes with phage start codons (ATG, GTG, and TTG) along with reformatting the gene model before formatting for display as a feature track for JBrowse. tRNA and tmRNA genes are predicted by **ARAGORN** v2.36 and also formatted for display as a feature track in JBrowse [51].

Phage genomes are highly compact due to packaging constraints imposed by the amount of genomic DNA or RNA that can fit into the capsid [52,53]. With a typical >90% coding density, protein-coding genes often overlap by several bases with adjacent coding sequences. Many overlapping genes are missed by gene calling algorithms trained on bacterial genomes which are not subjected to compaction [53–55], which is why the CPT Galaxy pipeline is designed to allow comparison of the results from three programs. MGA and GLIMMER results usually agree, and predict the majority of protein-coding genes in the genome. However, when gaps are present, they can often be filled by reading frames identified in the naïve (and noisy) GetORFs/Sixpack track. The user may evaluate the tool outputs and determine which predicted genes are promoted to protein-coding gene features in the Apollo editor window. As ARAGORN only displays tRNA predictions of high confidence, these are typically all promoted as features. The TransTermHP results are evaluated by the user, and may be promoted as annotations based on the criteria of a score >95, a hairpin stem with at least 5 matches, a minimum four-T run in the T-tail after the stem, and genomic context. These annotations can then be imported back into Galaxy as a GFF3 file for further analysis.

### Functional annotation workflow

**Fig 3:**
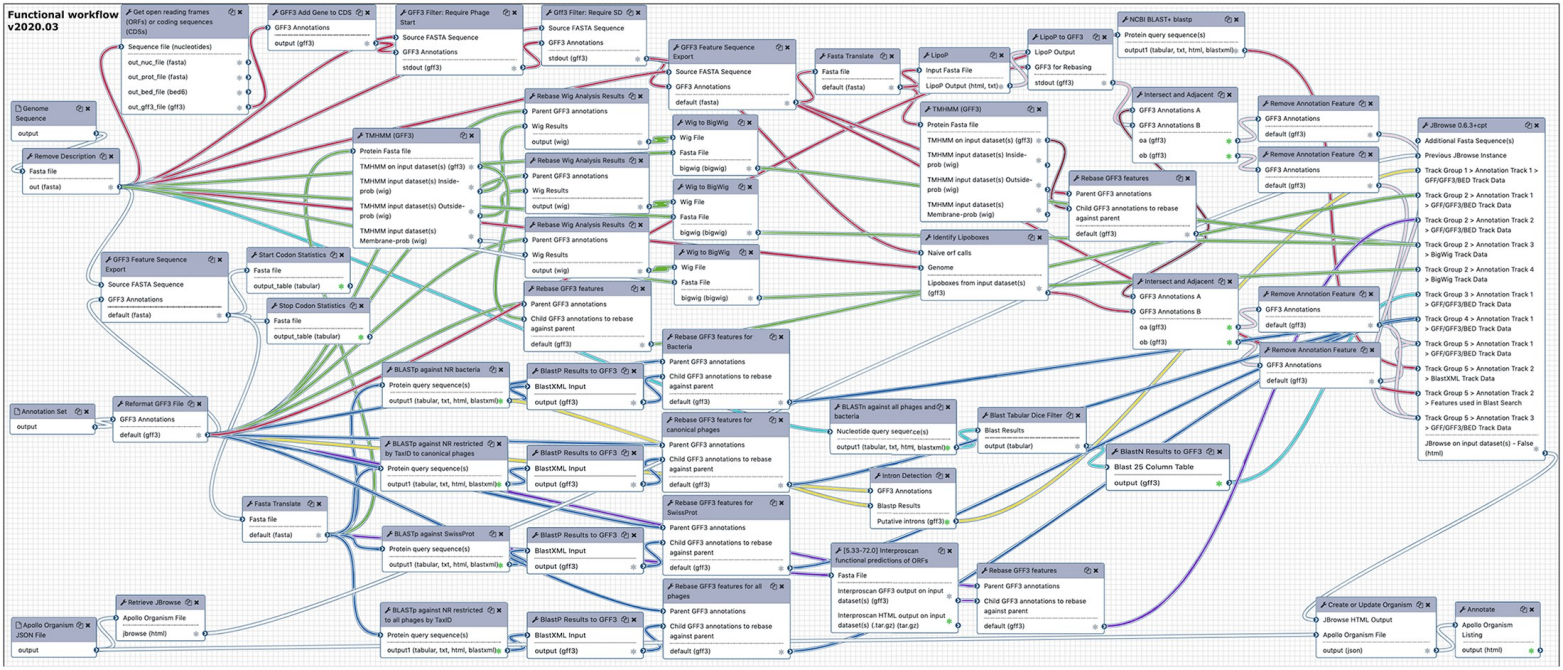
The functional workflow links tools in Galaxy used for functional prediction. Inputs for the functional workflow are gene calls paired with their genome. These are piped through five sub-paths within the analysis. 1) The BLASTn path uses full genomic nucleotide sequence (light blue noodles). 2) The BLASTp protein analysis against curated (UniProtKB SwissProt) and sequence-inclusive databases (NCBI nonredundant (nr)) (dark blue noodles). 3) The search for interrupted genes like introns compiles separate CDS hits to the same protein (yellow noodles). 4) A directed search for spanin proteins using TMHMM, lipobox-finding (using LipoP and a motif search), and BLASTp against a curated database (magenta and pink noodles). 5) Domain analysis plots comprehensive TMHMM outputs and InterProScan results for conserved domains and signatures (green and purple noodles, respectively).

The goal of the functional workflow is to provide the user with contextualized evidence to predict the function of the proteins encoded by their input genes. The 57 steps comprising the functional workflow are the main workhorse of the phage annotation process (Fig 3). Due to the use of BLAST and InterProScan, the functional workflow can take several hours to complete, commensurate with the size of the input dataset.

The independent segments of the workflow will be discussed in these sections: BLAST analysis, InterProScan, and transmembrane domain finding (the intron and spanin detection segments are described above). As an added value feature, start and stop codon statistics are calculated.

*BLAST*. First, the genome sequence is analyzed by BLASTn (dc-megablast) with a 0.001 expectation cutoff, against the NCBI nonredundant (nt) database restricted by all phages and bacterial TaxIDs. The tabular BLASTn results are parsed with the **Blast Tabular Dice Filter** tool (filters results with low Dice coefficient, and requires 50–100% identity), then mapped back onto the genome as a GFF3 for display in JBrowse. Second, the CDS features in GFF3 format are extracted from the structural annotation results, translated, and analyzed in four separate jobs by traditional BLASTp at a 0.001 expectation value cutoff and default parameters. One job searches against the UniProtKB SwissProt database, which includes only manually annotated and reviewed sequences, providing a first good clue to their function. Two BLASTp jobs search against the NCBI nr database restricted to all phages and canonical phages (S3 Table) by TaxID. The fourth BLASTp analysis searches against the entire nr database. All BLASTp outputs (in XML format) are converted to GFF3, then ‘rebased’, or mapped, back to the parent genome for display as an evidence track in JBrowse format.

*InterProScan* is a conserved domain search tool incorporating information from several other services [56–59]. In the CPT Galaxy workflow, the same translated CDS features used for BLASTp analysis are searched against conserved domain databases by InterProScan, which returns a GFF3-formatted result that is mapped (or ‘rebased’) back onto the parent genome for JBrowse display.

*Transmembrane domains* are predicted from the translated features using TMHMM 2.0 [28]. The simple output is a GFF3 file, which, when displayed in Apollo shows the location of the probable TMD, and gives its predicted orientation in the feature name. Three additional outputs for inside, outside, and membrane probability are converted from wig to bigwig format for a plot format display [60], and mapped back onto the parent GFF3 at the proper location. Additionally, transmembrane domains and signal peptides are predicted by Phobius, which is incorporated into the InterProScan software package [59,61].

The final output of the workflow is the direct Apollo link to the updated JBrowse instance for the organism. In Apollo, each evidence track will be displayed in context with the rest of the genome for evaluation in functional prediction. After predictions are completed, the genome annotations can be returned to Galaxy with the Retrieve Data tool, yielding a FASTA and paired GFF3 file. These can then be converted into the five-column table required by NCBI for deposition into Genbank, as described elsewhere.

### Comparative genomics workflow

**Fig 4:**
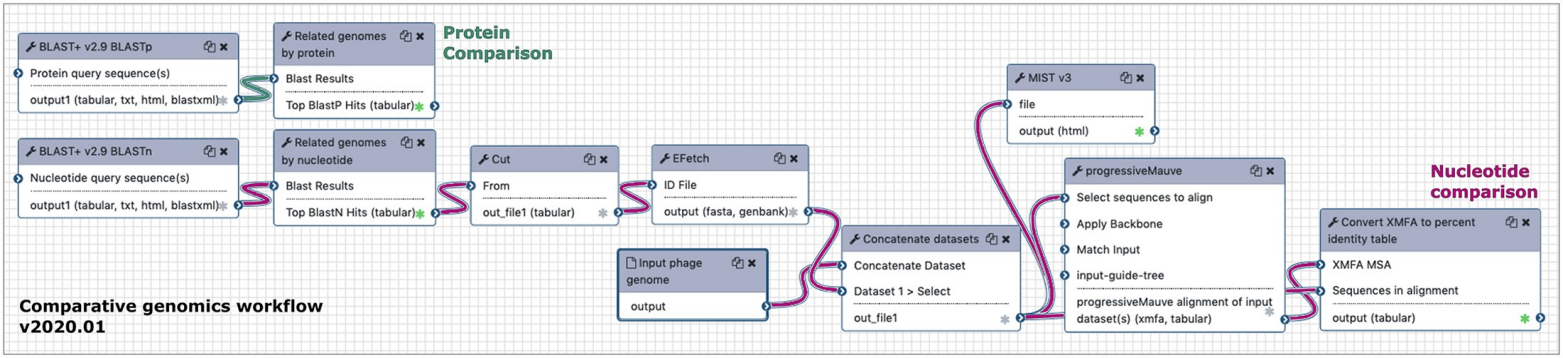
The comparative genomics workflow calculates nucleotide and protein similarity to other phages. The protein comparison branch starts with a BLASTp job against the nr database, restricted by all phage TaxIDs (see full list in S3 Table), and is then sorted according to the organisms with the highest number of unique protein hits (teal noodles). The nucleotide comparison branch begins with a BLASTn job against the nt database, also restricted by all phage TaxIDs. Top nucleotide hits are sorted based on dice score, which accounts for the total coverage. The top five genome sequences are fetched from NCBI, concatenated with the query genome and routed to MIST v3 for a dot plot, and progressiveMauve for calculation of pairwise percent identity (magenta noodles).

The goal of the comparative workflow is to identify the most related phages to the query organism, compared at both nucleotide and protein levels. This workflow requires the genomic DNA sequence in FASTA format and the GFF3 annotations of protein-coding genes, and returns tables listing the most related organisms by BLASTn and BLASTp analysis, and a series of pairwise DNA dot plots. There are 11 steps in the comparative workflow and it usually takes less than ten minutes to run (Fig 4).

Protein comparison is performed by a BLASTp search against NR restricted by all phage TaxIDs (S3 Table) and an expectation value cutoff of 0.001. The top hits (default is 20) are parsed from the custom tabular output by TaxID and returned as a table with the phage common name, NCBI TaxID, and number of similar unique proteins they share with the query phage.

Full-genome nucleotide comparison starts with a traditional BLASTn megablast restricted to all phages in the NCBI nr database using a 0.001 expectation value cutoff. The number of user-specified top hits (default is ten) are parsed from the custom tabular output based on percent identity (calculated for the query:subject pair according to the Dice coefficient = (2 * # identical matches)/((genome 1 length) + (genome 2 length))). With this information, the full FASTA record for each top matching genome is retrieved via the NCBI **EFetch** utility. After concatenation, the related genomes are processed by **MIST** v3 to generate a dot plot; MIST builds upon Gepard [62] to generate a matrix of multiple pairwise dot plots for a set of input sequences. The MIST Python script calls Gepard for each pairwise comparison and builds the combined image and image map. DNA sequences are also compared by **progressiveMauve** v2.4.0, with the aligned blocks extracted and output as a table with the **Convert XMFA to percent identity table** tool, which presents overall percent nucleotide identity as a Dice coefficient [62,63].

The data in these tables can be directly used for publication, as reference for finding literature on the genome type, or as the basis for additional comparative genomics including the generation of synteny plots using tools in CPT Galaxy like **X-Vis**, **Genome Mapper**, or **EasyFig** [64].

### More on workflows

The three workhorse workflows described can be customized in the Galaxy workflow editor by addition or removal of individual tools, updating tool functionality, or simply tweaking job parameters as needed. New mainstay workflow versions we prepare regularly become publicly available to the community, and users can contribute their customizations or specialized workflows as well. The signature workflows described above are geared towards the annotation of new genomes, but we will note a few additional applications of the entire interface here. Importantly, pre-annotated genomes can be loaded into Apollo using the *Upload Genbank into Apollo* (Genbank file input) or *Upload Previously Annotated Sequence to Apollo* (FASTA and GFF3 input) workflows for updating by the user or community, as determined by whom the user gives access to the organism. This will aid in our goal of reaching at least one “gold-standard” annotated genome per representative phage type. Users can also compare directly to the original annotations by adding them as an evidence track with the *GFF3 to Apollo evidence track* workflow.

CPT Galaxy users have also built and shared a variety of generally applicable routines. There is a whole collection of workflows that will perform an analysis and push the results to an evidence track in Apollo, usually BLAST jobs. The workflows *Custom BLASTp to Apollo Evidence Track–UserDB* and its variation from a *Local DB* allow running a BLASTp analysis against a user-generated local database. A specific use case is the *BLAST antiCRISPRdb to Apollo Evidence Track* workflow, which searches against a local copy of the curated anti-CRISPRdb [65]. Another useful workflow set, *X-vis from GFF3/Fasta*, will generate a synteny map converting BLAST similarity into protein XMFA format for visualization across the entire genome. The direct links to current versions of the workflows mentioned here are listed in S4 Table. A full list of the most recent published versions for the annotation pipeline and all other workflows can be accessed at https://cpt.tamu.edu/galaxy-pub/workflows/list_published.

## Results & discussion

### Comparison to other available annotation tools

Various groups host annotation systems publicly accessible for phage annotation. DNAMaster is arguably the most widely used program for teaching phage annotation in the undergraduate education context [66]. Thousands of students have performed phage annotation through the SEA-PHAGES program in this stand-alone Windows program written originally by Jeffrey Lawrence. Recently, an independent research group released multiPhATE, a downloadable command-line bioinformatics pipeline for functional annotation of phage isolates [9] One unique aspect in multiPhATE is an accompanying tool designed specifically to detect phage genes called PHANOTATE [54].

Prior to these examples with adaptations tuned to phage annotation, most automated pipelines in wide use were developed around prokaryotic annotation needs. The service offered with deposit to Genbank, the Prokaryotic Genome Annotation Pipeline (PGAP), is now available as a stand-alone program for bacterial and archaeal genomes and used for all RefSeq sequences [67,68]. DFAST is a downloadable and web-based prokaryotic genome annotation pipeline that boasts a 10-minute or less processing time for full bacterial genomes [69]. The RASTtk pipeline is a downloadable and web-based prokaryotic genome annotation service, used in the microbiology community as the basis for PATRIC (PAThosystems Resource Integration Center) [70–72]. Their use of subsystems to perform quick and automatic annotations has also been applied to phage genome annotation, recently described in manual format [73].

Given its widespread use, we compared the output of annotation with the tools in CPT Galaxy to RASTtk for five phages representing all the major morphotypes: four that we originally annotated, and phage T1 from RefSeq (S5 Table). The final number of genes called was similar, within six of each other, with CPT Galaxy annotations always having the higher count. The phages annotated using the CPT Galaxy-Apollo platform had fewer proteins without a functional prediction (hypothetical or phage protein). Finally, the number of genes called with a valid Shine-Dalgarno sequence was higher for CPT Galaxy, but only by a maximum of seven genes in total. In addition to missing more complex genome features, such as terminal repeat regions and rho-independent terminators, RASTtk did not specifically assign function to various known phage proteins with bioinformatically recognizable characteristics, such as the spanins and translationally frameshifted tape measure protein chaperones.

No currently available automated annotation algorithm can reasonably be expected to reach the highest quality product that manual, human effort can produce, and that in turn is inferior to empirical data to support annotations. However, it is not always feasible to invest significant researcher time on upstream annotation processes. While experienced annotators can be quite efficient, when speed is of the essence, automated pipelines can be combined with the convenient browsing and editing tools available in the CPT Galaxy for polishing. Additionally, the annotation can be performed collaboratively across groups or institutions, making use of the “Google Docs”-type functionality, as we have done in several instances [74–76]. Finally, the system’s application to a classroom setting is also immediately apparent, even a virtual classroom, where teams can work on the same genome, and all contributions are logged per user.

## Conclusion

Here, we have described a complete, easy-to-use web-based platform for phage annotation. The powerful suite of tools housed in the CPT Galaxy instance provides a dynamic, scalable framework for genome data analyses that can be used independently of, or in conjunction with the community genome editor, Apollo. In both research and educational contexts, the CPT Galaxy and Apollo system allows users to focus on the biology behind annotation rather than the minutiae of server maintenance, command-line operations and file management. This is essential to allow us to use the flood of sequencing data to move deeper into the understanding of phage biology and the fundamental principles that govern their bacterial hosts, and make informed decisions about their applications in the future.

## Supporting information

S1 Table A list of all tools used in the structural (S), functional (F), and comparative genomics (C) workflows. The current working versions of the tools presented here are available at https://github.com/TAMU-CPT/galaxy-tools.
(XLSX)

S2 Table The consensus and derivative possible Shine-Dalgarno sequences that are the default in ShineFind. By default, the ShineFind tool searches within 3–24 nucleotides upstream of a given start codon for the longest, or first, possible Shine-Dalgarno sites that match to the following, starting with the *E*. *coli* consensus.
(XLSX)

S3 Table List of common names and identifiers used in BLAST. Each category may be used separately or together to restrict a BLAST analysis by TaxID. Phage common names and NCBI accession numbers are included in the custom canonical phages database, which is also pre-compiled as a BLAST database. Bolded rows contain the well-studied representatives of each morphotype.
(XLSX)

S4 Table Helpful CPT Galaxy workflow links. Current versions of all published workflows can be accessed at https://cpt.tamu.edu/galaxy-pub/workflows/list_published.
(XLSX)

S5 Table Comparison of phage genome annotations performed using different methods. Five phages, the canonical phage T1 and four previously annotated at the CPT, were assessed with RASTtk and features were compared. The number of gene features listed is strictly a count, not reflecting the possibility that completely different genes, or genes with alternate starts, were called. The hypothetical protein count is meant to be a proxy for functional annotation. Since RASTtk also uses the nonspecific name “phage protein”, this term was separately tallied. In the additional features row are listed genes and other aspects of phage genomes that are typically only identified by manual inspection, and which only our pipeline identifies here. tmp = tape measure protein, and terminators refer to rho-independent terminators predicted by TransTermHP. Compiled annotation data can be browsed by the public at https://cpt.tamu.edu/apollo/jbrowse/.
(XLSX)
